# Dataset of DNA methylation profiles of 189 pediatric central nervous system, soft tissue, and bone tumors

**DOI:** 10.1016/j.dib.2024.110590

**Published:** 2024-06-07

**Authors:** Robin Jugas, Petra Pokorna, Sona Adamcova, Katerina Kozelkova, Dana Knoflickova, Hana Palova, Jaroslav Sterba, Ondrej Slaby

**Affiliations:** a Department of Biology, Faculty of Medicine and Central European Institute of Technology, Masaryk University, Kamenice 753/5, 625 00 Brno, Czechia; b Department of Biochemistry, Faculty of Science, Masaryk University, Kamenice 753/5, 625 00 Brno, Czechia; c Department of Oncological Pathology, Masaryk Memorial Cancer Institute, Žlutý kopec 543/7, 656 53 Brno, Czechia; d Department of Pediatric Oncology, University Hospital Brno and Faculty of Medicine, Masaryk University, Jihlavská 20, 625 00 Brno, Czechia

**Keywords:** Pediatric oncology, Epigenetics, Targeted methylation sequencing, Precision medicine

## Abstract

Alterations in DNA methylation profiles belong to important mechanisms in cancer development, and their assessment can be utilized for rapid and precise diagnostics. Therefore, establishing datasets of methylation profiles can improve and deepen our understanding of the role of epigenetic changes in cancer development as well as improve our diagnostic capabilities. In this dataset, we generated NGS data for 189 samples of pediatric CNS, soft tissue, and bone tumors. The sequencing libraries were prepared using methyl capture bisulfite sequencing, an effective compromise between whole-genome bisulfite sequencing and array-based methods with a more limited scope of target regions. The larger part of the cohort was processed with the Agilent SureSelectXT Human Methyl-Seq kit (149 samples) and the rest with the Illumina TruSeq Methyl Capture EPIC Library Prep Kit (40 samples). The data presented in this article may help other researchers further elucidate the importance of methylation in diagnosing pediatric CNS tumors, soft tissue, and bone tumors.

Specifications Table

**Table:** 

Subject	Cancer Research
Specific subject area	DNA methylation profiles of pediatric CNS, soft tissue, and bone tumor samples
Data format	Raw
Type of data	.fastq.gz files (raw sequencing data)
Data collection	DNA isolated from frozen tumor tissue samples was used for library preparation utilizing hybridization-based target enrichment and subsequent bisulfite conversion. Two different kits/panels were used, including Agilent SureSelectXT Human Methyl-Seq and Illumina TruSeq Methyl Capture EPIC Library Prep Kit. Libraries were sequenced on the Illumina NextSeq 500 device, sequencing reads were quality-checked, and raw sequencing reads were deposited to The European Genome-phenome Archive.
Data source location	Institution: Central European Institute of Technology, Masaryk University City/Town/Region: Brno Country: Czech Republic Latitude and longitude for collected samples/data: 49.17893945357473, 16.570828322685344
Data accessibility	Repository name: The European Genome-phenome Archive Data identification number: https://doi.org/10.5281/zenodo.10877204 Direct URL to data: https://ega-archive.org/studies/EGAS50000000051 Instructions for accessing these data: Please reach out to the contact person of the Data Access Committee to be granted permission to download the data.

## 1 Value of the Data

• This dataset contains methyl capture bisulfite sequencing data of 189 pediatric CNS, soft tissue, and bone tumors, enabling single-base resolution methylation detection of approximately 3.2 million or 4.2 million CpGs for libraries prepared with the Agilent kit and the Illumina kit, respectively.
• The cohort represents various types of pediatric CNS, soft tissue, and bone tumors, including some rare diagnostic units underrepresented in other cancer datasets.
• The dataset can be integrated into a large cohort for machine learning training and validation in cancer research.

## 2 Background

Aberrant DNA methylation belongs to important cancer hallmarks and is particularly pertinent in the context of pediatric tumors, which are characterized by a low number of somatic alterations and in which epigenetic mechanisms are increasingly understood to play a crucial role in carcinogenesis [1]. Cancer-specific methylation patterns have recently emerged as a powerful and robust approach in cancer diagnostics, enabling diagnosis refinement of major diagnostic groups such as tumors of the central nervous system (CNS) [2,3] and, more recently, sarcomas [4]. Furthermore, methylation profiling has been acknowledged in the last 5th edition of the WHO Classification of CNS tumors, which validates the role of molecular findings in establishing a precise cancer diagnosis. The advancements in the field of methylation-based classification of tumors can be further facilitated by the development of novel machine-learning tools, which, however, require collecting large amounts of samples.

Apart from diagnostics, the study of cancer-associated methylation patterns offers opportunities for a better understanding of tumor biology and ongoing research into effective therapies. A staple methodologic approach used for large-scale methylation profiling has been the use of microarray platforms [5]. Nonetheless, the next-generation sequencing (NGS) approach has been becoming more prevalent, owing primarily to the increasing accessibility of NGS platforms in both research and routine laboratories. Thus, we performed targeted methylation sequencing for 189 samples of pediatric CNS, soft tissue, and bone tumors. Part of this dataset has been used for the development of a cross-platform neural network-based framework for tumor classification [6].

## 3 Data Description

The dataset [7] is assigned to one EGA study and consists of three separate datasets. The separate datasets are named Agilent CNS cohort (EGAD50000000072, 97 samples of CNS tumors), Agilent Sarcoma cohort (EGAD50000000073, 52 samples of soft tissue and bone tumors), and Illumina cohort (EGAD50000000074, 40 samples consisting of 39 CNS tumors and 1 sarcoma sample). The data files are provided as pairs of raw fastq files corresponding to the respective sequencing libraries prepared through targeted methylation sequencing, which employs target enrichment designed to capture CpG islands with adjacent areas, gene promoters, and some known DMRs and bisulfite conversion. The content of each dataset in terms of included diagnostic units is briefly outlined in Table 1, with more details provided in Supplementary Tables 1–3. Supplementary Tables 1–3 contain information on the diagnosis, age, sex, and disease stage of all samples included in the respective datasets.

**Table 1:** Overview of diagnosis representation in cohorts.

Agilent CNS cohort	
**Total number of samples**	97
**Diagnosis groups**	
Medulloblastoma	32
Posterior fossa ependymoma	18
Diffuse midline glioma, H3 K27-altered	10
Pilocytic astrocytoma	10
Atypical teratoid/rhabdoid tumor	6
Supratentorial ependymoma	5
Astroblastoma, MN1-altered	4
Diffuse pediatric-type high-grade glioma, H3-wildtype and IDH-wildtype	4
Others	8
**Agilent Sarcoma cohort**	
**Total number of samples**	52
**Diagnosis groups**	
Ewing sarcoma	12
Osteosarcoma	9
Alveolar rhabdomyosarcoma	7
Embryonal rhabdomyosarcoma	4
Undifferentiated round cell sarcomas with CIC or BCOR alteration	4
Synovial sarcoma	3
Others	13
**Illumina cohort**	
**Total number of samples**	40
**Diagnosis groups**	
Medulloblastoma	15
Posterior fossa ependymoma	8
Diffuse midline glioma, H3 K27-altered	5
Others	12

## 4 Experimental Design, Materials and Methods

### 4.1 Sample collection and DNA extraction

The cohort comprises tumor samples of pediatric, adolescent, and young adult patients of Czech descent collected at the Department of Pediatric Oncology, University Hospital Brno, Czech Republic. Selected demographic data are listed in Supplementary Table 1. Tumor tissue was obtained during surgery, which was performed as a part of routine diagnostic and/or therapeutic management. It was subsequently frozen and stored at −80 °C and later used for DNA extraction. The extraction was carried out using mechanic homogenization with ceramic beads and subsequent column-based extraction with DNeasy Blood & Tissue Kit (Qiagen). DNA purity was assessed with NanoDrop 2000c Spectrophotometer (ThermoFisher Scientific), and the precise quantity was determined using Qubit dsDNA BR Assay Kit (ThermoFisher Scientific).

### 4.2 Library preparation and sequencing

Sequencing libraries were prepared with either TruSeq Methyl Capture EPIC Library Prep Kit (Illumina) or SureSelectXT Methyl-Seq Library Preparation Kit combined with SureSelectXT Human Methyl-Seq target enrichment panel (Agilent). For the TruSeq Methyl Capture EPIC Library Prep Kit, 500 ng of input DNA was used, whereas, for the SureSelectXT Methyl-Seq Library Preparation Kit, the starting DNA amount was 1 µg. Both library preparation procedures followed the manufacturer's instructions. Sequencing libraries prepared with TruSeq Methyl Capture EPIC Library Prep Kit were sequenced on the NextSeq 500 device using NextSeq 500/550 Mid Output Kit v2.5 (150 Cycles) (Illumina) in a paired-end setting of 2 x 80 bp. Libraries prepared with SureSelectXT Methyl-Seq Library Preparation Kit were also sequenced on the NextSeq 500 device using either NextSeq 500/550 Mid Output Kit v2.5 (300 cycles) or NextSeq 500/550 Mid Output Kit v2.5 (150 Cycles) in a paired-end setting of 2 × 151 bp and 2 × 80 bp, respectively.

### 4.3 Data quality assessment

The raw sequencing reads were quality-checked with the FastQC v0.11.9 [8]. Adapters and low-quality 3‘ ends were trimmed with Trim Galore [9]. The Bismark methylation toolkit v0.23.1 was used to align and call the methylation loci [10]. The human reference hg19/GRch37 was used. All the following analyses and visualizations were done in R 4.3.1. The methylation processing pipeline used is stored in the following repository: “github.com/robinjugas/PipelineForEpigeneticProfiling.”

**Fig. 1:**
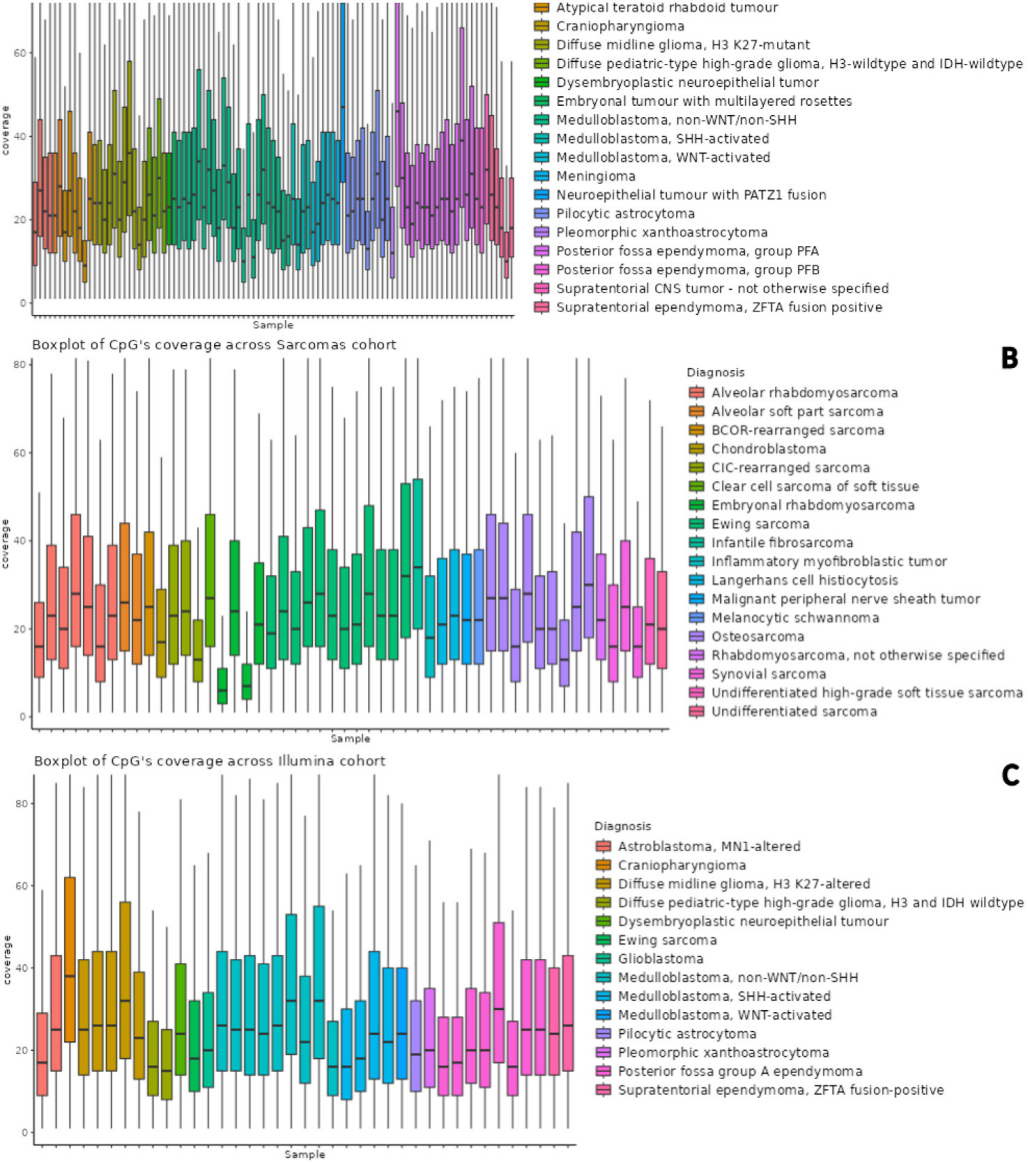
Boxplots of CpG's reads coverage A. Agilent CNS cohort B. Agilent Sarcoma cohort. C. Illumina cohort. The samples are colored according to their tumor diagnosis.

**Fig. 2:**
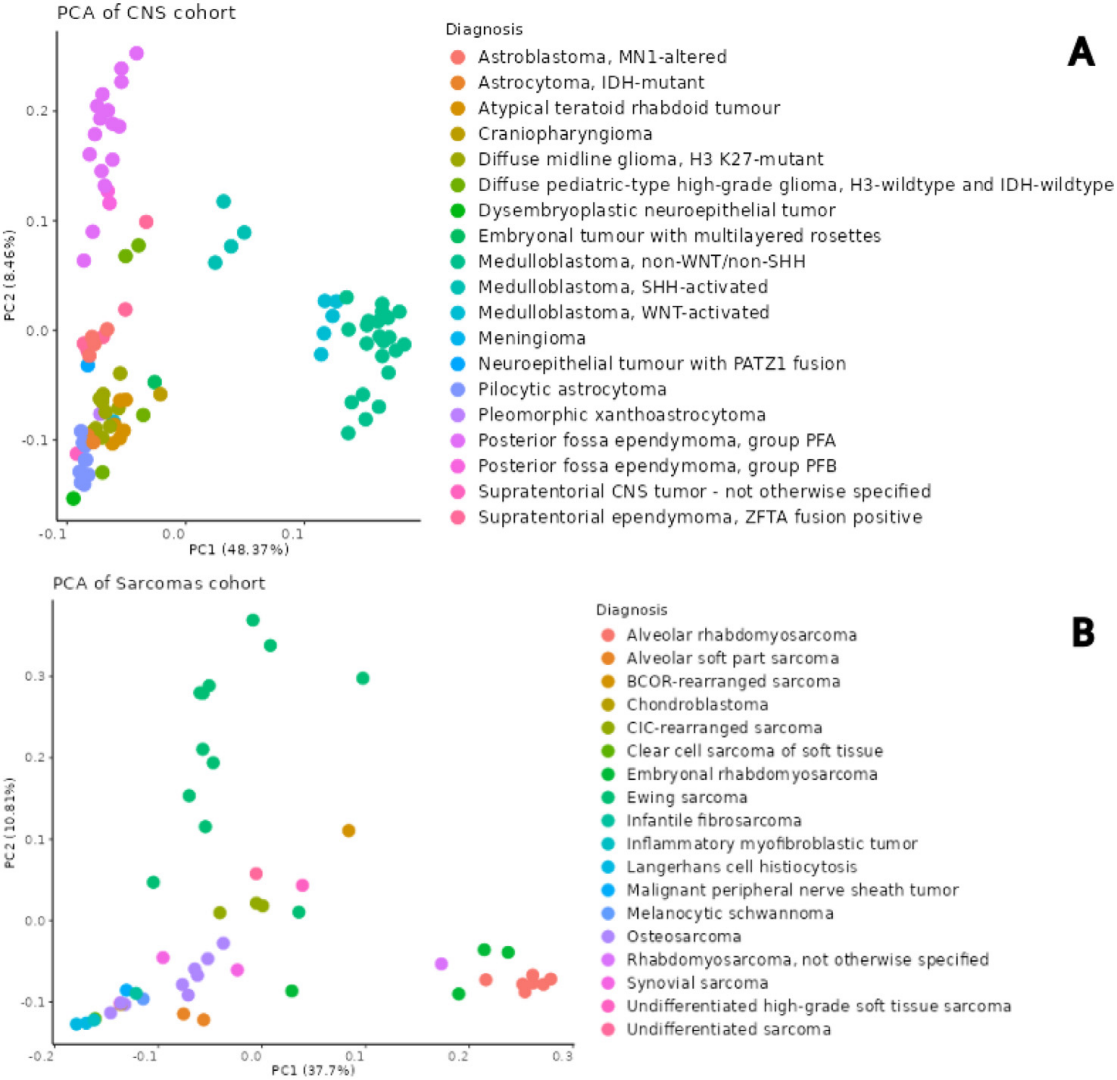
The PCA plots of Agilent Methyl-Seq cohorts with color-highlighted diagnoses **A**. CNS cohort **B**. Sarcomas cohort.

The mean coverage of all CpGs was 13× across the dataset. We focused on CpGs covered by more than 5 reads, as generally used. The coverage at these CpG loci per each sample is depicted in bar charts in Fig. 1. The number of CpGs with coverage higher than 5× was approximately 3.1 million and 4.2 million for libraries prepared with the Agilent kit and the Illumina kit, respectively. The PCA analysis performed on ten thousand most variable shared CpGs with coverage higher than 5× showcased a concordance between methylation profile and diagnosis in all separate datasets (see Fig. 2).

## Limitations

The presented datasets include a diverse range of diagnoses. However, compared to the typical distribution observed in CNS tumors, there is an increased prevalence of embryonal and ependymal tumors. This is primarily due to the significant role of methylation profiling in assessing diagnostic subgroups. From a technical standpoint, the two capture panels used in this study target different ranges of CpGs, which needs to be taken into account in downstream analyses when working with the entire dataset.

## Ethics Statement

Informed consent was obtained from all subjects prior to sample processing. The research was carried out in accordance with the Declaration of Helsinki and approved by the Masaryk University Ethical Committee (approval no. 15/2018).

## CRediT authorship contribution statement

**Robin Jugas:** Formal analysis, Data curation, Visualization, Writing – original draft. **Petra Pokorna:** Methodology, Investigation, Writing – review & editing. **Sona Adamcova:** Methodology, Investigation. **Katerina Kozelkova:** Methodology, Investigation. **Dana Knoflickova:** Methodology, Investigation. **Hana Palova:** Methodology, Investigation. **Jaroslav Sterba:** Conceptualization, Supervision, Funding acquisition, Writing – review & editing. **Ondrej Slaby:** Conceptualization, Supervision, Funding acquisition, Writing – review & editing.

## Data Availability

Dataset of DNA methylation profiles of 189 pediatric central nervous system, soft tissue, and bone tumors (Original data) (The European Genome-phenome Archive)

## Appendix Supplementary materials

Image, application 1
